# Glucose Lowering Therapeutic Strategies for Type 2 Diabetic Patients with Chronic Kidney Disease in Primary Care Setting in France: A Cross-Sectional Study

**DOI:** 10.1155/2013/640632

**Published:** 2013-04-04

**Authors:** N. Grandfils, B. Detournay, C. Attali, D. Joly, D. Simon, B. Vergès, M. Toussi, Y. Briand, O. Delaitre

**Affiliations:** ^1^IMS Health France, 5-7 Place de la Pyramide, 92088 Paris La Défense, France; ^2^Cemka-Eval, 43 Boulevard du Maréchal Joffre, 92340 Bourg-la-Reine, France; ^3^Medical office, 4 Rue de L'Ile de France, 91860 Epinay sous Senart, France; ^4^Nephrology Department, Hôpital Necker-Enfants Malades, 149 Rue de Sèvres, 75015 Paris, France; ^5^Diabetes Department, Hôpital de la Pitié-Salpétrière, 47 Boulevard de l'Hôpital, 75013 Paris, France; ^6^INSERM CESP, U-1018, Villejuif, 94800, France; ^7^Endocrinology Department, Hôpital du Bocage, 1 Boulevard Jeanne d'Arc, 21079 Dijon, France; ^8^Boehringer-Ingelheim France, 14 Rue Jean Antoine de Baïf, 75013 Paris, France

## Abstract

*Aim*. To understand glucose lowering therapeutic strategies of French general practitioners (GPs) in the management of type 2 diabetes mellitus (T2DM) patients with chronic kidney disease (CKD). *Methods*. A multicenter cross-sectional study was conducted from March to June 2011 among a sample of French GPs who contribute to the IMS Lifelink Disease Analyzer database. Eligible patients were those with T2DM and moderate-to-severe CKD who visited their GPs at least once during the study period. Data were collected through electronic medical records and an additional questionnaire. *Results*. 116 GPs included 297 patients: 86 with stage 3a (Group 1, GFR = 45–60 mL/min/1.73 m^2^) and 211 with stages 3b, 4, or 5 (Group 2, GFR < 45 mL/min/1.73 m^2^). Patients' mean age was approximately 75 years. Insulin was used in 19% of patients, and was predominant in those with severe CKD. More than two-thirds of patients were treated with glucose lowering agents which were either contraindicated or not recommended for CKD. Conclusion Physicians most commonly considered the severity of diabetes and not CKD in their therapeutic decision making, exposing patients to potential iatrogenic risks. The recent patient oriented approach and individualization of glycemic objectives according to patient profile rather than standard HbA1c would improve this situation.

## 1. Introduction

Type 2 diabetes mellitus (T2DM) is a major health problem with a steady increase of prevalence worldwide. Data from the 2011 National Diabetes Statistics (National Institutes of Health) report that 8.3% of the US population had diabetes [1]. In France, the prevalence of pharmacologically treated diabetes increased from 4.39% in 2009 to 4.64% in 2011 according to the data collected by the French National Sickness Fund [2, 3]. Around one-third of patients with T2DM manifest some form of clinical kidney damage and patients have a progressively increasing risk of developing chronic kidney disease (CKD) [4, 5]. Conversely, for 45% of patients who receive dialysis, diabetes is the primary cause of kidney failure [6–8]. In France, diabetes along with hypertension constitute the primary cause of kidney failure [9].

CKD is a common disease with an increasing prevalence, partially as a consequence of increasing prevalence of diabetes. According to the American National Kidney Foundation (NKF), renal function is chronically altered when anomalies in markers of renal disease (clinical proteinuria, haematuria, leukocyturia, morphological and histological anomalies, or markers of tubule dysfunction) persist for more than three months or glomerular filtration rate (GFR) is less than 60 mL/min/1.73 m^2^ for more than three months [10]. The disease is progressive and the definition of its staging differs in the French (HAS) and the American (Kidney/Disease Outcomes Quality Initiative, NKF 2007) guidelines [10, 11]. The French Nephrology Society (FNS) has tried to harmonize the different recommendations by proposing the classification in 6 stages, grouped by kidney function as described by the GFR. Patients with stage 1 or 2 CKD (GFR > 90 mL/min/1.73 m^2^ and GFR = 60–90 mL/min/1.73 m^2^, resp.) have normal or mildly reduced kidney function, those with stages 3a and 3b (GFR = 45–60 mL/min/1.73 m^2^ and GFR = 30–45 mL/min/1.73 m^2^) have moderately reduced kidney function, and those with stage 4 or 5 (GFR = 15–30 mL/min/1.73 m^2^ and GFR ≤ 15 mL/min/1.73 m^2^), respectively, have severe or end-stage renal disease.

Moderate-to-severe CKD (GFR < 60 mL/min/1.73 m^2^) is observed in 20%–30% of patients with T2DM [12, 13]. The presence of both diabetes and CKD obliges clinicians to take into account several clinical factors in the management of these diseases in order to (1) achieve glycaemic control, (2) avoid progression and/or complications of renal disease, and (3) control the risk of cardiovascular events and premature mortality which is extremely high in this category of patients [14].

The epidemiology of CKD among diabetic patients in France is relatively well known. For example, data from the 2007 ENTRED cohort [15] showed that two-thirds of T2DM patients had low GFR values: 14% with CKD stage 3a and 8% with CKD stages 3b, 4, or 5. Among this latter group, the proportion of people aged over 75 years and of women is significantly higher than that in the populations of T2DM patients with lower stages of CKD.

The management of diabetic patients with CKD in primary care is less well known. Understanding of therapeutic strategies used by general practitioners for these patients is important and allows shaping of appropriate educational messages towards physicians for a better management of T2DM patients with CKD.

The aim of BEMEDIR (medical need, diabetes and renal failure (BEsoin MEdical, Diabète et Insuffisance Rénale)) study was to describe and analyze how T2DM patients with moderate-to-severe CKD are managed by GPs in France. 

## 2. Materials and Methods

We conducted a multicenter cross-sectional study in primary care setting in France. GPs contributing to the panel of IMS Lifelink EMR Disease Analyzer (DA) were invited to participate in the study. DA is a database of longitudinal electronic medical records (EMRs) of about 5 million patients collected from a panel of about 1200 physicians since 2000. Its validity and representativeness have been analyzed and published previously [16]. The study population consisted of patients diagnosed with T2DM and moderate-to-severe CKD whose EMR data were available in DA and whose GPs accepted to complete an additional questionnaire on their diabetes care. In our study, each participating physician was asked to include up to four T2DM patients with CKD, two with CKD at stage 3a (Group 1, with GFR = 45–60 mL/min/1.73 m^2^), and two with CKD at stage 3b, 4, or 5 (Group 2, with GFR < 45 mL/min/1.73 m^2^). In addition to the information available through the patients' EMRs, an additional questionnaire was completed by physicians for each patient to provide more details on the management of diabetes and renal disease, their satisfaction with glycaemic control of the patients, the reasons for their treatment choices, and so forth. This additional questionnaire was then linked to the EMRs using GPs' unique national number, patient's date of birth and sex, and the date of the visit. An independent scientific committee validated the study protocol and questionnaire.

Once the data collection was complete, the database was locked for analysis. The GFR of each patient was calculated post hoc using MDRD formula to validate the classification of patients into Group 1 and 2 provided by the GPs.

## 3. Results

### 3.1. Patients Characteristics

A total of 116 GPs participated in the study (participation rate 10%). They included a total of 375 patients from 1 March 2011 to 15 June, 2011. Among these, 45 patients were excluded from the study because their EMR did not contain sufficient data to allow patient category control with GFR calculation. Of the remaining 330 patients, GPs had included 167 in Group 1, 152 in Group 2, and 11 without mentioning the group. After post hoc control of GFR value for each patient, 86 patients (29%) were included in Group 1 and 211 (71%) in Group 2. Finally, 33 patients were excluded from analysis as their GFR was more than 60 mL/min/1.73 m^2^ and therefore failed to meet the inclusion criteria. The remaining 297 patients were used for further analysis.

Characteristics of each group of patients are shown in Table 1. Patients were generally of older age with a mean age of approximately 75 years, are overweight with a mean BMI of 29 kg/m^2^, and had a relatively long (mean 12–14 years) history of diabetes. The mean HbA1c was approximately 7.1% in both groups. Group 1 patients were predominantly males, while Group 2 patients were predominantly females. 

### 3.2. Glucose Lowering Treatments

**Figure 1:**
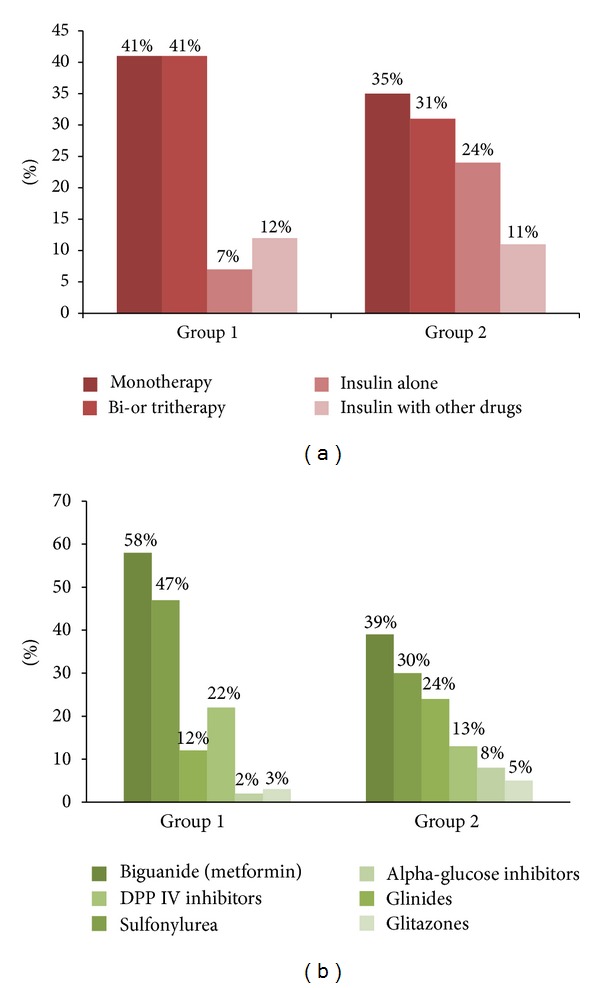
Glucose lowering therapy strategies adopted in the 195 patients with available prescription data. A large proportion of patients were treated with oral mono, dual, or triple therapies while the percentage of patients treated with insulin alone or in association with other drugs was lower and was higher in patients with severe CKD compared to moderate ones.

Analysis of the type of glucose lowering therapy was performed in 195 of the 297 included patients who had available data on drug prescriptions. As shown in Figure 1(a), a large proportion of diabetic patients with CKD were treated with oral glucose lowering agents. In patients with moderate CKD (Group 1), 81% were treated with oral therapy, while 7% received insulin alone and 12% received insulin in association with oral therapy. Among patients with more severe kidney dysfunction (Group 2), a higher number were treated with insulin (24% alone and 11% in combination with oral agents). With regard to the choice of glucose lowering drugs (Figure 1(b)), 47% were treated with sulfonylurea and 58% were treated with metformin in Group 1, while these proportions were 30% and 39%, respectively, in Group 2. Correspondingly, a minority of patients were prescribed glitazones, alpha glucosidase inhibitors, or glinides (2, to 12% in Group 1 and 5, to 24% in Group 2). 

### 3.3. Factors Taken into Account by GPs in the Choice of Glucose Lowering Strategy

**Figure 2:**
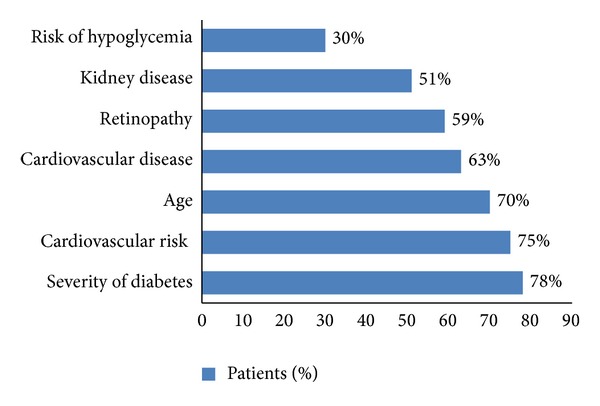
Factors considered by GPs when selecting glucose lowering therapy in CKD patients. Among the top five factors that GPs took into account, severity of diabetes was the most frequently mentioned while only one GP out of three mentioned the risk of hypoglycaemic episodes.

GPs were asked to select the top five factors influencing their choices when determining a glucose lowering treatment strategy in their CKD patients. Factors the GPs have taken into consideration are reported in Figure 2. Severity of diabetes was the most frequently mentioned factor (78%), while only one GP out of two mentioned CKD (51%) among the first five factors considered. It is worth noting that only one GP out of three (30%) mentioned the risk of hypoglycemic episodes, even though this category of patients is at high risk of this event.

### 3.4. Adequacy of Glucose Lowering Therapy for CKD and Glycaemic Control

GPs were asked whether they were satisfied with glycaemic control achieved for each patient. For 73% of patients, GPs declared being satisfied with the level of glycaemic control achieved. Mean HbA1c level was lower among patients whose GPs were satisfied with their glycaemic control (Table 2). The mean HbA1c level for patients with satisfactory glycaemic control (as declared by the GPs) was 6.7% (6.9% and 6.7% in Group 1 and 2, resp.) and ranged from 6.6% for patients receiving oral monotherapy to 7.4% for patients receiving insulin. Mean HbA1c in patients for whom GPs were not satisfied was approximately 8.2% in the two groups.

A 63% of patients were treated with glucose lowering treatments which were either contraindicated or not recommended for CKD patients based on the current French guidelines and summaries of product characteristics of prescribed drugs. A 45% of patients had satisfactory glycemic control, while 18% did not have satisfactory glycemic control based on the opinion of the GP. Only 37% of patients were treated with safe glucose lowering drugs for CKD. A 25% of them had satisfactory glycemic control based on the GPs opinion (Table 3).

### 3.5. Changes in Glucose Lowering Therapy Choice

In 57% of patients, therapeutic strategies had not been changed by GPs during the previous year, with no difference between the two groups (Table 4). The achievement of successful glycemic control was considered the main reason. Other reasons included, insufficient follow-up time to check for the effects of lifestyle and dietary measures (18.4%), waiting for specialist advice (13.5%), risk of hypoglycemia (6.7%), of side effects (6.1%) or of CKD worsening (6.7%), and taking into account patient insulin injection acceptance (7.4%). However, when GPs did introduce changes in glucose lowering therapies, they did so in relation to the presence of CKD in 55% of patients in Group 1 and 62% in Group 2. The most frequent change concerned the replacement of the oral glucose lowering drug with another (28% in Group 1 and 19% in Group 2). Other common changes were withdrawal of a drug (14% and 22%, resp.), dose reduction (21% and 17%, resp.), and a switch to insulin (21% and 22%, resp.).

When GPs were asked to provide their opinion on the best therapeutic strategy they would recommend in order to meet glycaemic control objectives in T2DM patients with CKD, most of them favored adopting a strict compliance with lifestyle and dietary measures (protein restriction, smoking cessation, physical activity, etc.) as the most fundamental aspects which should be taken into consideration. One GP out of three mentioned the need for drugs that could be used without restriction among patients with CKD (Table 5).

## 4. Discussion

GPs participating in this study had difficulty in appropriately identifying and classifying patients with CKD. This could lead to suboptimal therapeutic strategy, that is, lack of consideration of the severity of renal impairment in glucose lowering treatment strategy. 

However, GPs' therapeutic management of T2DM patients with CKD was guided by the general glycaemic control achievement (HbA1c threshold), and they often ignored the severity of renal dysfunction (i.e., in selecting more appropriate treatments and HbA1c targets). GPs' satisfaction with patient glycaemic objectives, as well as their therapeutic strategy, was closely associated with HbA1c value of ~7%. Only half of the GPs mentioned the presence of CKD as one of the five most important factors influencing their strategy, and even fewer mentioned the risk of hypoglycemic episodes which is particularly important in patients with renal impairment.

For over half of patients, GPs did not change their glucose lowering therapy during the last year, thus potentially exposing CKD patients to risk of adverse drug reactions. However, when such change was conducted, they declared that renal disease was the main motivation for it.

Like any other observational study, our study was limited by the availability of data provided by its investigators. The 10% participation rate of GPs was similar to other studies conducted by the same team using a randomized list of physicians. However, we did not compare characteristics of participating and nonparticipating physicians. We had to exclude about 15% of patients because of unavailability of appropriate variables allowing the post hoc calculation of GFR. However, we consider that this choice was crucial in avoiding classification and inclusion bias. In other patients where these data were available, the calculation of GFR allowed reclassification of patients in Groups 1 and 2 and the exclusion of about 10% of noneligible patients.

The observed difficulties of GPs in managing T2DM patients with CKD may be linked to the lack of specific French guidelines on the management of diabetic patients with renal disease at the time of the study. Also, for some glucose lowering drugs, such as metformin, there is no consensus on the acceptable renal threshold for continuing the same therapy in case of renal dysfunction [17]. 

The presence of kidney disease brings an additional layer of complexity to the management of T2DM patients, compared to those with diabetes alone. As the kidneys play an important role in the elimination of insulin and some oral glucose lowering drugs, impaired renal function makes CKD patients exposed to drugs or their metabolites for a longer period of time, potentially resulting in adverse side effects [18]. This includes a higher risk of hypoglycemia to which the reduction of renal neoglucogenesis associated with CKD largely contributes. Therefore, glucose levels of diabetic patients with CKD must be closely monitored. This often results in the adjustment of glucose lowering therapy. Moreover, T2DM patients with CKD are often older in age compared to the diabetic patients without CKD, often have other cardiovascular risk factors [19] and are at higher risk of developing cardiovascular disease, polypharmacy, and drug interactions.

Our study provides new qualitative information on physician's priorities and decision-making process in the management of T2DM patients with CKD. It also confirms the results of another published research on glucose lowering strategies used by GPs for these patients [15], showing that metformin and sulfonylurea were the most prescribed glucose lowering agents. The authors of the latter study explained that the presence of kidney disease which, in some cases, remained unidentified did not significantly influence the prescribing of glucose lowering agents.

In France, a group of experts from the French Nephrology Society and the Diabetes Society developed recommendations to guide professionals in the management of diabetic patients with impaired renal function [20]. According to these recommendations, metformin remains the first-line drug for the management of T2DM patients, but its dosage must be divided by two when GFR is between 60 and 30 mL/min/1.73 m^2^ and it must be withdrawn in patients with GFR below 30 mL/min/1.73 m^2^. The use of sulfonylurea is possible but the predominant renal elimination of this class should prompt the clinicians to favor products with short half lives and inactive metabolites and to adopt a strict dosage adjustment. Glucose lowering therapy with insulin is recommended in cases of renal impairment and the importance of the dose adjustment should be emphasized. These recommendations are in line with the most recent American and European guidelines (ADA/EASD) on the management of T2DM patients [21–23] which suggest individualization of glycaemic objectives based on patient's conditions, with more ambitious individual HbA1c goals in younger patients among those with CKD. Metformin should be used with caution among patients with mild-to-moderate CKD because of its renal elimination. Although there is debate on the threshold of serum creatinine or GFR, it should be avoided in severe CKD patients (GFR < 30 mL/min/1.73 m^2^). Most insulin secretagogues, especially glibenclamide, should be avoided in patients with CKD, because of the risk of hypoglycaemia. Most dipeptidyl peptidase-4 (DPP-4) inhibitors have prominent renal elimination, and thus dose reduction is necessary among patients with CKD for sitagliptin, vildagliptin, and saxagliptin. The exception is linagliptin which is mainly eliminated unchanged in bile and intestine. Therefore, for this product, no dosage regimen adjustment is necessary based on its summary of product characteristics for a renal impaired patient, regardless of the severity of the renal impairment. 

## 5. Conclusions

We identified a number of issues regarding the management of T2DM patients with moderate-to-severe CKD by French GPs. Our study suggests that GPs are mainly focused on managing the glycaemic control of their patients, and they do not always consider CKD as a coexisting condition in the management of these patients. These findings reinforce the need for more accurate information for GPs about T2DM and CKD. 

Implementation of specific guidelines for T2DM patients with CKD would allow GPs to be better informed and to adapt their therapeutic strategy to each clinical situation. As CKD patients are generally of older age, at high risk of developing cardiovascular disease and have other comorbid conditions, the patient centered approach and individualization of therapeutic strategy based on comorbidities, as introduced by the new ADA/EASD guidelines would improve this situation. 

## Figures and Tables

**Table 1:** Patient demographics and disease characteristics.

Characteristics	Group 1	Group 2
Age in years	*N* = 86	*N = *211
Mean (SD)	76.2 (±11.3)	75.8 (±10.5)
Gender ratio	*N = * 86	*N = *211
(M/F)	1.9	0.7
BMI (kg/m²)	*N = * 86	*N = *207
Mean (SD)	28.2 (±4.8)	29.3 (±5.5)
BMI (kg/m²): *N* patients (%) with	*N = * 86	*N = *211
BMI < 25	25 (29%)	44 (21%)
25 ≤ BMI < 30	35 (41%)	81 (39%)
BMI ≥ 30	26 (30%)	82 (40%)
GFR (mL/min/1.73 m²)	*N = * 86	*N = *211
Mean (SD)	51.9 (±4.3)	29.41 (±13.0)
Time since CKD diagnosis: *N* patients (%)		
≥1 year	*N = *85 66 (77%),	*N = *207 175 (83%)
≤1 year	*N = *84 20 (23%)	*N = *203 36 (17%)
Proteinuria: *N* patients (%) with		
Microproteinuria	39 (46%)	124 (60%)
Macroproteinuria	7 (8%)	38 (19%)
Time since diabetes diagnosis (years)	*N = *86	*N = *2011
Mean (SD)	12.6 (±8.4)	14.2 (±9.0)
HbA1c	*N = *76	*N = *197
Percentage (SD)	7.2 (±1.1)	7.1 (±1.3)
HbA1c < 7%	*N = *76	*N = *197
*N* patients (%)	36 (47%)	106 (54%)

*N*:number of patients, BMI: body mass index; CKD: chronic kidney disease; GFR: glomerular filtration rate; SD: standard deviation.

**Table 2:** Association of HbA1c values with GP satisfaction of glycaemic control and with treatment types among all included patients (*N* = 268).

	Mean HbA1c % (SE)*
GP satisfied with glycaemic control	6.7 (0.9)
Treatment prescribed	
Monotherapy	6.6 (0.6)
Bitherapy	6.9 (0.8)
Tritherapy	7.0 (1.4)
Insulin + oral anti-diabetic	7.2 (1.2)
Insulin	7.4 (2.7)
Group	
Group 1 (*N = * 75)	6.9 (0.9)
Group 2 (*N = *193)	6.7 (0.9)
GP not satisfied with glycaemic control	8.2 (1.4)
Group	
Group 1 (*N = * 75)	8.3 (1.3)
Group 2 (*N = *193)	8.2 (1.5)

*Based on last measure of HbA1c. SE: standard error.

**Table 3:** GP's satisfaction with glycaemic control and its association with indication or recommendation of glucose lowering therapy in CKD patients.

	Treatment with contraindicated or not recommended drugs		
GP satisfied with glycaemic control	Yes (%)	No (%)	All (%)
Yes (%)	81 (45)	44 (24)	125 (69)
No (%)	33 (18)	22 (12)	55 (30)

All (%)	114 (63)	66 (36)	180 (100)

**Table 4:** Change of treatment over the past year by stage of CKD among all included patients.

Change of treatment over the past year by stage of CKD	Group 1 (%)	Group 2 (%)	All (%)
Yes	36 (42)	93 (44)	129 (43)
No	50 (58)	118 (56)	168 (57)
All	86 (100)	211 (100)	297 (100)

**Table 5:** GP's optimal solution to meet glycaemic control objectives.

GP's optimal solution to meet glycaemic control objectives	Patients % *N* = 78
Patients' strict respect of lifestyle and dietary measures (including smoking cessation)	67
Regular physical exercise	46
New drug without contraindications for CKD	29
Patient acceptance to switch to insulin	28
Improvement in treatment observance	21
Strengthening of therapeutic education programs	17
